# Impact of dapagliflozin on left ventricular diastolic function of patients with type 2 diabetic mellitus with chronic heart failure

**DOI:** 10.1186/s12933-018-0775-z

**Published:** 2018-10-08

**Authors:** Fumitaka Soga, Hidekazu Tanaka, Kazuhiro Tatsumi, Yasuhide Mochizuki, Hiroyuki Sano, Hiromi Toki, Kensuke Matsumoto, Junya Shite, Hideyuki Takaoka, Tomofumi Doi, Ken-ichi Hirata

**Affiliations:** 1 Division of Cardiovascular Medicine, Department of Internal Medicine, Kobe University Graduate School of Medicine, 7-5-2, Kusunoki-cho, Chuo-ku, Kobe, 650-0017 Japan; 2 Tatsumi Clinic, Himeji, Japan; 3 Division of Cardiology, Osaka Saiseikai Nakatsu Hospital, Osaka, Japan; 4 Division of Cardiology, Aijinkai Takatsuki Hospital, Takatsuki, Japan; 5 Division of Cardiology, Kobe Red Cross Hospital, Kobe, Japan

**Keywords:** Sodium glucose cotransporter type 2 inhibitors, Type 2 diabetes mellitus, Left ventricular diastolic function, Heart failure

## Abstract

**Background:**

The objective of this study was to investigate the impact of sodium glucose cotransporter type 2 (SGLT2) inhibitors on left ventricular (LV) diastolic function of type 2 diabetes mellitus (T2DM) patients with heart failure (HF).

**Methods:**

This trial was a prospective multicenter study of 58 T2DM patients with stable HF at five institutions in Japan. Patients who had been taking at least one antidiabetic drugs other than SGLT2 inhibitors started the administration of 5 mg/day of dapagliflozin. The physical examinations, blood tests, and echocardiography were performed at baseline and 6 months after administration of dapagliflozin. The primary endpoint was defined as a change in mitral inflow E and mitral e′ annular velocities (E/e′) between baseline and 6 months after the administration of dapagliflozin. The secondary end points consisted of a change in brain natriuretic peptide (BNP), LV mass index (LVMI) and left atrial volume index (LAVI).

**Results:**

E/e′ significantly decreased from 9.3 to 8.5 cm/s (p = 0.020) 6 months after administration of dapagliflozin. LAVI and LVMI significantly decreased from 31 to 26 mL/m^2^ (p = 0.001), and from 75.0 to 67.0 g/m^2^ (p < 0.001), respectively, 6 months after administration of dapagliflozin. No significant change was observed in BNP (from 27.9 to 28.9 pg/mL; p = 0.132) 6 months after administration of dapagliflozin, except for a significant decrease from 168.8 to 114.3 pg/mL (p = 0.012) in patients with BNP ≥ 100 pg/mL.

**Conclusion:**

This prospective multicenter trial showed the beneficial effect of SGLT2 inhibitors on LV diastolic functional parameters for T2DM patients with HF. Our findings may thus offer a new insight into the management of T2DM patients.

*Trial registration* UMIN000019789, Registered 28 September 2014, Date of registration: 11/14/2015, Date of enrolment of the first participant to the trial: 6/15/2016, Date of enrolment of the last participant to the trial: 12/9/2017

## Introduction

Type 2 diabetes mellitus (T2DM) is a major cause of heart failure (HF), both with reduced ejection fraction (HFrEF) and with preserved ejection fraction (HFpEF), as well as cardiovascular disease [1, 2]. Sodium glucose cotransporter type 2 (SGLT2) inhibitors are a new class of anti-hyperglycemic agents for T2DM, which act insulin independently to selectively inhibit renal glucose reabsorption, thereby increasing urinary glucose excretion [3]. Large, rigorously conducted clinical trials using an SGLT2 inhibitor, such as empagliflozin for the EMPA-REG OUTCOME trial [4] and canagliflozin for the CANVAS Program, [5] found that patients with T2DM at high risk of cardiovascular events derived cardiovascular benefits from the SGLT2 inhibitor as compared with from a placebo. In addition, SGLT2 inhibitors have the potential to improve cardiovascular risk profiles, including lower blood pressure and weight loss, as well as to reduce glycated hemoglobin levels in T2DM patients [4–7]. Findings of a large clinical trial to evaluate the effects of dapagliflozin, another SGLT2 inhibitor, on cardiovascular outcomes, DECLARE TIMI-58 [8], have not been published yet. This trial is being conducted with a broad range of T2DM patients with either established cardiovascular disease or multiple cardiovascular risk factors. In other studies, left ventricular (LV) diastolic function was found to be strongly associated with HFpEF, or possibly with HF with mid-range ejection fraction (HFmrEF) [9]. In addition, LV diastolic dysfunction was identified as an independent predictor of outcome even for patients with HFrEF [9, 10]. Interest has therefore been growing in the utility of SGLT2 inhibitors for the improvement of cardiovascular outcomes for T2DM patients with high risk of cardiovascular events as a potential means for better management of T2DM patients. However, the impact of SGLT2 inhibitors including dapagliflozin on the LV diastolic function of T2DM patients with HF remains unclear.

The objective of this study was, therefore, to investigate the impact of dapagliflozin on the LV diastolic function of T2DM patients with stable HF.

## Methods

This trial was a prospective multicenter study to investigate the effect of SGLT2 inhibitors (dapagliflozin) on LV diastolic functional parameters of T2DM patients with stable HF at five institutions in Japan. The trial was registered with the University Hospital Medical Information Network (UMIN) Clinical Trials Registry (registration number UMIN000019789), and conforms to the principles outlined in the Declaration of Helsinki.

### Study population

Participants enrolled in this prospective trial were 58 T2DM patients with stable HF who had been taking at least one antidiabetic drugs other than SGLT2 inhibitors for more than 1 year between December 2015 and March 2016 at the participating centers. All patients had a previous history of HF, but they were in clinically stable condition at the time of enrollment, defined as an exacerbation of HF symptoms for at least 6 months. Patients were excluded from enrolment study if they met any of the following criteria: (1) age < 20 and > 75 years; (2) type I DM; (3) T2DM with HbA1c < 6.5% and > 10.0%; (4) insulin-dependent T2DM; (5) serious renal dysfunction defined as glomerular filtration rate < 45 mL/min/1.73 m^2^; (6) hypotension < 90/50 mmHg; (7) malignancy; (8) poor nutritional status; and (9) atrial fibrillation. The diagnosis of T2DM was based on the World Health Organization criteria [11]. According to the current guideline [10], patients were subsequently categorized as HFrEF, HFpEF or HFmrEF if their LVEF was < 40%, ≥ 50% or 40–49%, respectively. The protocol was approved by the institutional review board at each of the participating institutions, and written informed consent was obtained from all patients.

### Study protocol

Stable HF patients who had been taking at least one antidiabetic drugs other than SGLT2 inhibitors and who had consented to their participation in this study, started the administration of dapagliflozin of 5 mg/day. Other drugs were not changed after the start of administration of dapagliflozin. The physical examinations and blood tests were performed at baseline, 3 months, and 6 months after administration of dapagliflozin. Echocardiography was performed at baseline and 6 months after administration of dapagliflozin. Only if the patient’s HbA1c had failed to improve by 3 months after administration of dapagliflozin, the dapagliflozin dose was raised from 5 mg/day to 10 mg mg/day.

### Echocardiographic examination

Echocardiography was performed with commercially available ultrasound systems comprising Aplio Artida, Aplio 400 and Xario (Canon Medical Systems, Tochigi, Japan), Vivid E9 (GE-Vingmed, Horten, Norway) and iE33 and EPIQ7 (Philips Medical Systems, Andover, MA) [12]. Standard echocardiographic measurements were obtained in accordance with the current guidelines of the American Society of Echocardiography/European Association of Cardiovascular Imaging [13]. Specifically, the early diastolic (E) and atrial wave (A) velocities and the E-wave deceleration time were measured by means of pulsed wave Doppler recording from the apical four-chamber view. Spectral pulsed-wave Doppler-derived early diastolic velocity (e′) was obtained by averaging the septal and lateral mitral annulus, and the E/e′ ratio was calculated to obtain an estimate of LV filling pressure. LV mass was estimated by using the formula proposed by Devereux et al. [14], and the LV mass index (LVMI) was calculated by dividing LV mass by body surface area. LA volume was measured with the biplane Simpson’s method from the apical two-and four-chamber views, and the LA volume index (LAVI) was calculated by dividing LA volume by body surface area.

### Definitions of end point

The primary end point was defined as a change in E/e′ between baseline and 6 months after the start of administration of dapagliflozin. The secondary end points comprised a change in brain natriuretic peptide (BNP), LVMI or LAVI between baseline and 6 months after the start of administration of dapagliflozin.

### Statistical analysis

Continuous variables were expressed as mean values and standard deviation for normally distributed data, and as the median and interquartile range for non-normally distributed data. Categorical variables were expressed as frequencies and percentages. Paired t tests or Wilcoxon signed-rank test were used for group comparison between baseline and 6 months after the start of administration of dapagliflozin. For all steps, a p value of < 0.05 was regarded as statistically significant. All analyses were performed with commercially available software (SPSS software version 24.0, SPSS Inc., Chicago, IL).

## Results

### Patient characteristics

Five initially eligible patients (8.6%) were excluded from all subsequent analyses because of lost follow-up prior to 6 months follow-up, so that the final study group consisted of 53 patients. The baseline clinical and echocardiographic characteristics of the 53 T2DM patients are summarized in Table 1. Their mean age was 68 years (60–73), LV ejection fraction (LVEF) was 62.3% (49.3–68.3, lowest: 20%, highest: 81%), and 21 patients (38%) were female. HFpEF was observed in 37 patients (69%), and the remaining 16 patients (31%) were classified as HFmrEF or HFrEF. In addition, 50 patients (94%) of patients were in New York Heart Association functional class I.

**Table 1:** Baseline characteristics of patients

Clinical characteristics	
Age, years	68 (60–73)
Gender (female), n (%)	21 (38)
DM duration, years	7.0 (5.0–11.5)
Body weight, kg	66.5 (56.8–76.9)
BSA, m^2^	1.73 (1.57–1.90)
Systolic blood pressure, mmHg	130 ± 16
Heart rate, bpm	71 ± 12
BNP, pg/mL	27.9 (9.0–58.2)
BUN, mg/dL	14.7 (12.1–19.0)
Creatinine, mg/dL	0.80 ± 0.20
eGFR, mL/min/1.73 m^2^	70.6 ± 17.0
HbA1c, %	6.9 (6.7–7.6)
Hemoglobin, g/dL	13.7 ± 1.7
Hematocrit, %	41 ± 4.7
Lipid profiles, mg/dL	
Triglycerides	127 (83.7–185)
LDL cholesterol	98 (86–118)
HDL cholesterol	51.5 (45.0–60.2)
Uric acid, mg/dL	5.1 (4.4–5.9)
HF classification, n (%)	
HFpEF	37 (69)
HFrEF	7 (13)
HFmrEF	9 (17)
Comorbidities, n (%)	
Hypertension	43 (81)
Dyslipidemia	42 (79)
Cardiovascular event	12 (21)
Medications, n (%)	
CCB	19 (36)
ACEI/ARB	42 (79)
β-Blocker	27 (51)
Diuretics	10 (19)
Statin	37 (70)
Antidiabetic drugs	
DPP-4I	40 (75)
GLP-1 RA	1 (2)
SU	11 (21)
α-GI	9 (17)
Thiazolidinedione	11 (21)
Metformin	14 (26)
Echocardiographic parameters	
LV end-diastolic volume, mL	74.2 (55.1–104.1)
LV end-systolic volume, mL	24.7 (17.0–54.5)
LVEF, %	62.3 (49.3–68.3)
LVMI, g/m^2^	75.0 (61.7–92.0)
LAVI, mL/m^2^	31 (23–45)
e′, cm/s	6.36 ± 1.73
E/e′	9.3 (7.7–11.8)
E, cm/s	58.1 (46.8–70.9)
A, cm/s	76.1 ± 17.8
E/A	0.71 (0.6–0.80)

Data are mean ± SD for normally distributed data and median and interquartile range for non-normally distributed data, or n (%)

*DM* diabetes mellitus, *BSA* body surface area, *BNP* plasma brain natriuretic peptide, *LDL* low density lipoprotein, *HDL* high density lipoprotein, *HFpEF* heart failure with preserved ejection fraction, *HFrEF* heart failure with reduced ejection fraction, *HFmrEF* heart failure with mid-range ejection fraction, *CCB* calcium channel blocker, *ACEI* angiotensin-converting enzyme inhibitor, *ARB* angiotensin II receptor blocker, *DPP-4I* dipeptidyl peptidase-4 inhibitor, *GLP-1 RA* glucagon-like peptide-1 receptors agonists, *SU* sulfonylureas, *α-GI* α-glucosidase inhibitors, *LVMI* left ventricular mass index, *LVEF* left ventricular ejection fraction, *LAVI* left atrial volume index, *E* peak early diastolic mitral flow velocity, *e*′ spectral pulsed-wave Doppler-derived early diastolic velocity from the septal mitral annulus

### Comparison of patient characteristics at baseline and 6 months after administration of dapagliflozin

The clinical and echocardiographic characteristics of the 53 T2DM patients at baseline and 6 months after administration of dapagliflozin are summarized in Table 2. Body weight from 66.5 kg (56.8–76.9) to 63.9 kg (56.2–75.6) (p < 0.001), respectively. HbA1c and systolic blood pressure tended to decrease 6 months after administration of dapagliflozin from 7.2 ± 0.8% at baseline to 7.0 ± 0.8% (p = 0.108) and from 130 ± 16 to 128 ± 18 mmHg (p = 0.218), but the differences were not statistically significant. Furthermore, hemoglobin, hematocrit and high-density lipoprotein cholesterol showed significant increase from 13.7 ± 1.7 to 14.4 ± 1.6 g/dL (p = 0.020), from 41.0 ± 4.7 to 43.8 ± 4.5% (p = 0.002) and from 51.5 mg/dL (45.0–60.2) to 52 mg/dL (42.8–65.0) (p = 0.049) 6 months after administration of dapagliflozin.

**Table 2:** Comparison of variables between baseline and 6 months after the administration of dapagliflozin

	Baseline	6 months	*p* value
Clinical characteristics			
Body weight, kg	66.5 (56.8–76.9)	63.9 (56.2–75.6)	< 0.001
BSA, m^2^	1.73 (1.57–1.90)	1.72 (1.55–1.88)	< 0.001
Systolic blood pressure, mmHg	130 ± 16	128 ± 18	0.218
Heart rate, bpm	71 ± 12	70 ± 11	0.610
BNP, pg/mL	27.9 (9.0–58.2)	28.9 (9.6–62.9)	0.132
BUN, mg/dL	14.7 (12.1–19.0)	17.2 (14.2–21.2)	< 0.001
Creatinine, mg/dL	0.80 ± 0.20	0.86 ± 0.23	< 0.001
eGFR, mL/min/1.73 m^2^	70.6 ± 17.0	65.6 ± 15.3	0.001
HbA1c, %	7.2 ± 0.8	7.0 ± 0.8	0.108
Hemoglobin, g/dL	13.7 ± 1.7	14.4 ± 1.6	0.020
Hematocrit, %	41.0 ± 4.7	43.8 ± 4.5	0.002
Lipid profiles, mg/dL			
Triglycerides	127 (84–185)	116 (82–176)	0.155
LDL cholesterol	98 (86–118)	106 (89–126)	0.078
HDL cholesterol	51.5 (45.0–60.2)	52 (42.8–65.0)	0.049
Uric acid, mg/dL	5.1 (4.4–5.9)	4.7 (4.2–5.7)	0.057
Echocardiographic parameters			
LV end-diastolic volume, mL	74.2 (55.1–104.1)	68.5 (54.8–93.8)	0.270
LV end-systolic volume, mL	24.7 (17.0–54.5)	20.5 (15.2–57.1)	0.105
LVEF, %	62.3 (49.3–68.3)	63.6 (55.3–71.0)	0.011
LVMI, g/m^2^	75.0 (61.7–92.0)	67.0 (55.0–81.9)	< 0.001
LAVI, mL/m^2^	31 (23–45)	26 (21–32)	0.001
e′, cm/s	6.36 ± 1.73	6.82 ± 1.88	0.031
E/e′	9.3 (7.7–11.8)	8.5 (6.6–10.7)	0.020
E, cm/s	58.1 (46.8–70.9)	55.1 (45.3–73.7)	0.682
A, cm/s	76.1 ± 17.8	75.6 ± 16.8	0.765
E/A	0.71 (0.6–0.80)	0.70 (0.62–0.81)	0.818

Data are mean ± SD for normally distributed data and median and interquartile range for non-normally distributed data, or n (%)

Abbreviation as in Table 1

### Primary end point

**Fig. 1:**
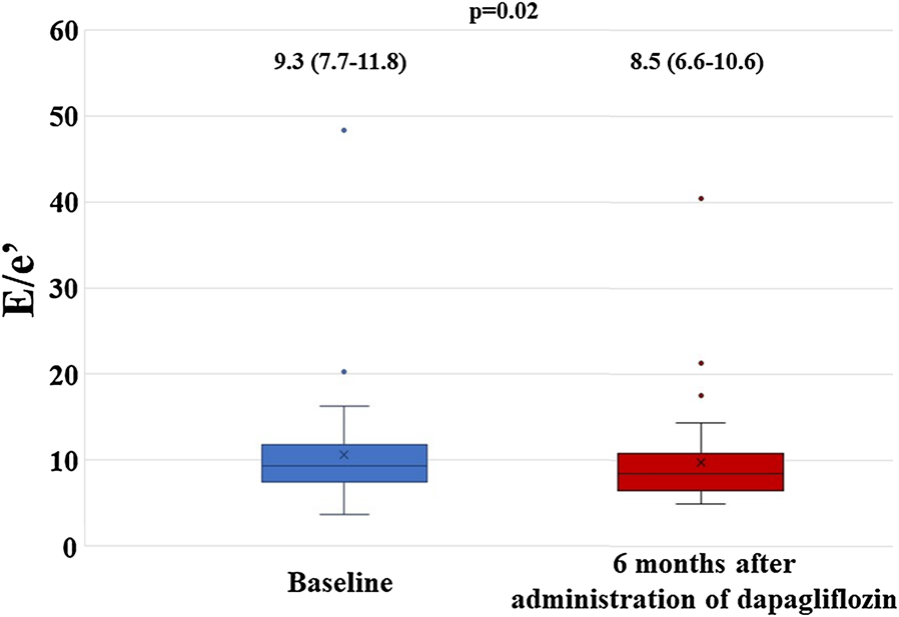
Result for primary end point, showing a significant decrease in E/e′ 6 months after administration of dapagliflozin

E/e′ showed significant decrease from 9.3 cm/s (7.7–11.8) to 8.5 cm/s (6.6–10.7) (p = 0.020) 6 months after administration of dapagliflozin (Fig. 1).

### Secondary end point

**Fig. 2:**
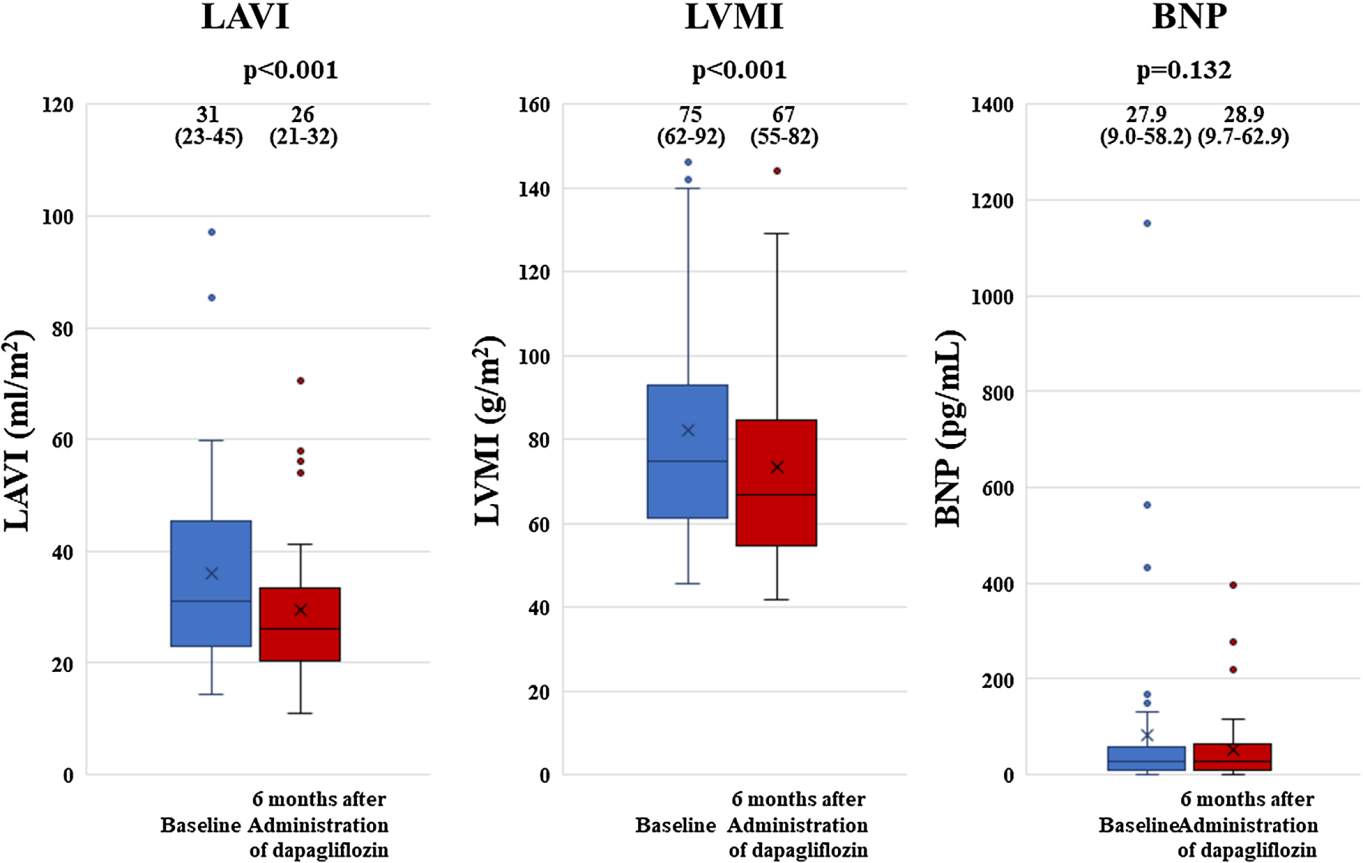
Results for secondary end points show a significant decrease in LAVI and LVMI significant decreased 6 months after administration of dapagliflozin. No significant change was observed in BNP 6 months after administration of dapagliflozin

**Fig. 3:**
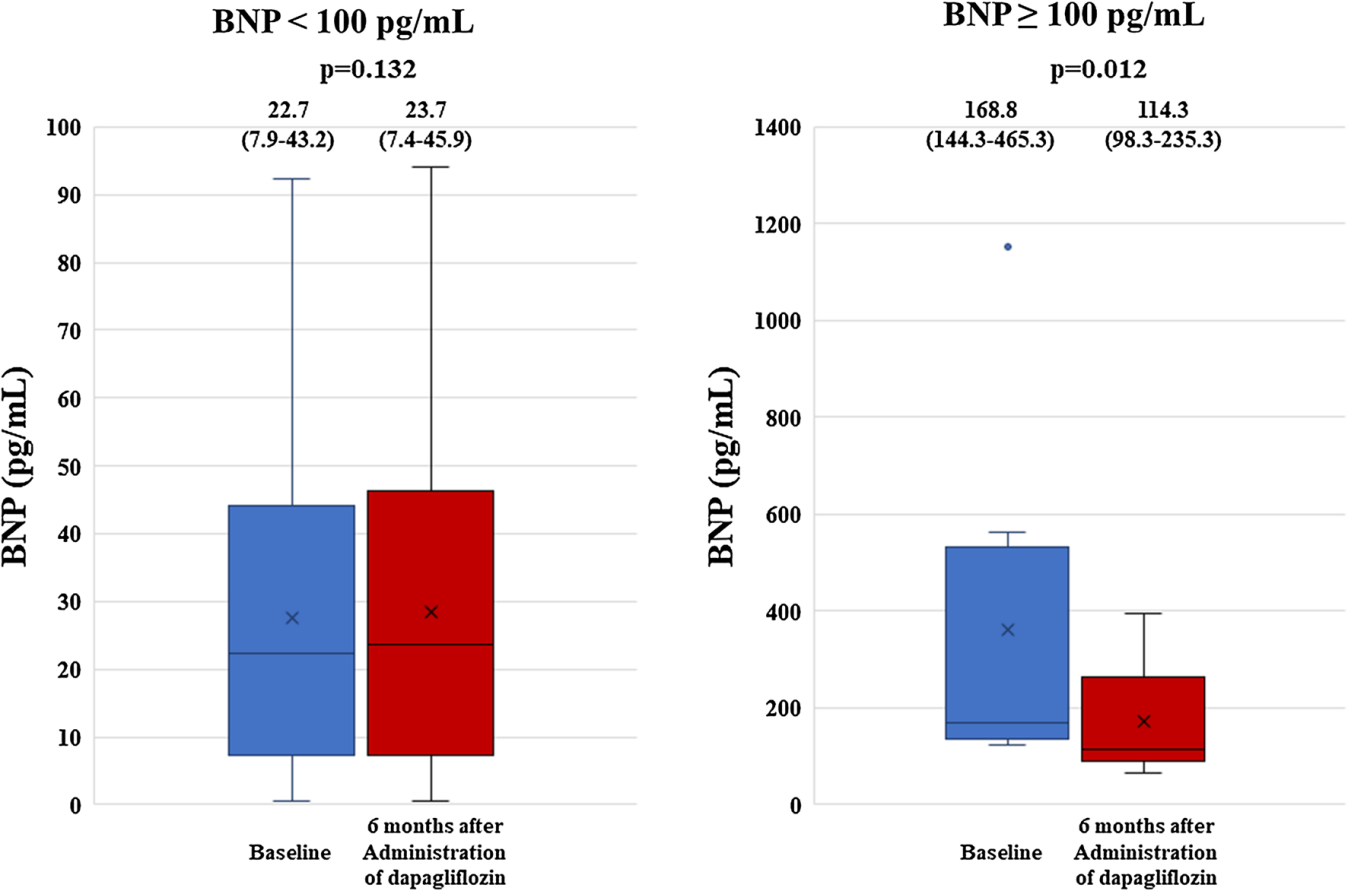
BNP decreased significantly in T2DM patients with BNP ≥ 100 pg/mL, but not in those with BNP < 100 pg/mL

The results for the secondary end point are shown in Fig. 2. LAVI and LVMI showed significant decreases from 31 mL/m^2^ (23–45) to 26 mL/m^2^ (21–32) (p = 0.001), and from 75.0 g/m^2^ (61.7–92.0) to 67.0 g/m^2^ (55.0–81.9) (p < 0.001) 6 months after administration of dapagliflozin, respectively (Fig. 2). No significant change was observed in BNP 6 months after administration of dapagliflozin from 27.9 pg/mL (9.0–58.2) at baseline to 28.9 pg/mL (9.6–62.9) (p = 0.132), but BNP significantly decreased from 168.8 pg/mL (144.3–465.3) to 114.3 pg/mL (98.3–235.3) (p = 0.012) in T2DM patients with BNP ≥ 100 pg/mL (Fig. 3).

## Discussion

The findings of our study indicate that LV diastolic function as assessed by E/e′ for T2DM patients with stable HF had significantly improved 6 months after administration of dapagliflozin. Other LV diastolic functional parameters such as LAVI and LVMI also improved 6 months after administration of dapagliflozin. In addition, BNP significantly decreased in T2DM patients with BNP ≥ 100 pg/mL.

### Characteristics of HF in T2DM

Heart failure can be defined as a complex clinical syndrome that results from any structural or functional impairment of ventricular filling or ejection of blood. Its prevalence increases with age and involves comorbidities such as hypertension, overweight/obesity, and T2DM. Structural cardiac changes seen in T2DM include increased interstitial fibrosis, increased LV wall thickness, and often increased LV mass [15], alterations which contribute to, but are not a prerequisite to, the development of functional myocardial impairments. As a consequence, LV diastolic dysfunction is the classical and most frequently observed early LV functional abnormality in T2DM patients [16], and asymptomatic LV diastolic dysfunction has been detected in up to 75% of normotensive DM patients without evident coronary artery disease [17]. The myocardial dysfunction in T2DM usually is progressive with an early asymptomatic phase where the heart hypertrophies, leading to LV diastolic dysfunction when LVEF has been preserved [18]. This is followed by a late stage, which is characterized by alteration in microvasculature compliance, an increase in LV size, and a decrease in cardiac performance leading to symptomatic HF. Predictors for progression to the late stage, which may take several years, include comorbidities often seen in T2DM such as coronary artery disease, hypertension, overweight/obesity, and microvascular changes [19].

### Impact of SGLT2 inhibitors on LV diastolic functional parameters

LV diastolic dysfunction is thought to be the underlying pathophysiological abnormality of patients with HFpEF and HFmrEF in particular, and thus its assessment plays an important role in diagnosis. In addition, it has been reported that LV diastolic dysfunction is independently associated with outcomes in patients with HFrEF as well as HFpEF or HFmrEF [9, 10]. SGLT2 inhibitors are associated with lower blood pressure and weight loss as well as a reduction in HbA1c levels, changes which in turn have a significant impact on LV function, so that SGLT2 inhibitors may ultimately have a potential beneficial effect on LV diastolic function in patients with T2DM. In addition, it has recently reported that SGLT2 inhibitors have a multifaceted effect on cardiac function including improvement in endothelial dysfunction and aortic stiffness [20], reduction in epicardial fat accumulation [21] as well as in visceral adipocyte hypertrophy [22]. Such effects may well lead to improvement in LV diastolic function. Verma et al. reported on the effects of the SGLT2 inhibitor empagliflozin (10 mg/day) 3 months after its initiation in terms of objective measurements of cardiac structure and function observed in 10 patients with T2DM and established cardiovascular disease. This short-term empagliflozin treatment was associated with a significant reduction in LVMI and improved LV diastolic function as assessed in terms of e′ [23]. More recently, Matsutani et al. performed a prospective single-center pilot study to evaluate the effects of canagliflozin on LV diastolic function in 37 T2DM patients with preserved LVEF [24]. They also found that LV diastolic function as assessed in terms of E/e′ and LVMI had significantly improved 3 months after the initiation of canagliflozin. Furthermore, multiple regression analysis showed that changes in HbA1c were an independent predictive factor for changes in E/e′. In line with previous studies involving T2DM patients without HF, our prospective multicenter trial using T2DM patients with HF showed that SGLT2 inhibitors were associated with improvements in LV diastolic functional parameters including E/e′, LAVI, and LVMI. Although the precise mechanism of the effect of SGLT2 inhibitors on LV diastolic function remains uncertain, the increased diuresis with reduced pre-load by means of SGLT2 inhibitors may play an important role. The average E/e′ at baseline in this study was relatively low because all patients were in stable HF condition. Since significant correlation was observed between E/e′ and pulmonary capillary wedge pressure in HF patients [25, 26], we believed that decreased E/e′ even in patients with normal range of E/e′ would be beneficial. In addition, BNP significantly decreased in T2DM patients with BNP ≥ 100 pg/mL in our study, so that SGLT2 inhibitors may have the potential to result in LV unloading in case of HF patients with an LV load at a certain level.

### Clinical implication

As mentioned earlier, the pathogenesis of DM-related cardiac dysfunction is thought to be multifactorial, and possibly a key factor for the development of HFpEF in T2DM patients, which presents as LV diastolic dysfunction. Moreover, LV diastolic function plays an important role for patients with HFrEF and HFmrEF as well as HFpEF in the development of cardiovascular events and outcomes. HFrEF has evolved into a distinct therapeutic entity partly because large outcome trials demonstrated the efficacy of neurohumoral inhibition, but no similar evolution has occurred in the case of HFpEF, because large trials testing neurohumoral inhibition consistently failed to attain a positive primary outcome [27–29]. In trials testing angiotensin-converting enzyme inhibitors, angiotensin II receptor blockers, β-blockers, or mineralocorticoid receptor antagonists, a modest positive trend was sometimes observed but only for secondary outcomes or retrospectively defined sub-groups [29–31]. Currently, therefore, there is no effective treatment for LV diastolic dysfunction to improve outcomes for HF patients. Our study, however, showed that LV diastolic functional parameters including E/e′, LAVI and LVMI for T2DM patients with stable HF had significantly improved 6 months after administration of dapagliflozin. Thus, our findings may well have clinical implications for better management of T2DM patients with HF.

### Study limitations

This study covered a relatively small number of patients without placebo-controlled group, so that further prospective studies with larger patient populations including placebo-controlled group will be needed to validate our findings. According to previous studies, SGLT2 inhibitors have the potential to result in lower blood pressure and weight loss for T2DM patients, in addition to reducing their HbA1c levels. Body weight significantly improved 6 months after administration of dapagliflozin, while HbA1c and blood pressure tended to decrease, but without statistical significance. The exact reason why SGLT2 inhibitors led to minor improvements in HbA1c and blood pressure remains unclear, but the heterogeneity of the study population of different HF classifications or small number of patients may be related to this finding. Furthermore, Habibi et al. showed that the treatment with the SGLT2 inhibitors (empagliflozin) improved LV diastolic function in obese female mice with diabetes, even in the absence of a reduction in blood pressure and HbA1c [32]. They also showed that the improvement in LV diastolic function was associated, not only with improved glycemia and blood pressure, but with improvements in cardiac structure, including reductions in interstitial myocardial fibrosis and associated pro-fibrotic SGK1/ENaC protein expression levels, cardiomyocyte hypertrophy and cardiomyocyte mitochondrial ultrastructure.

## Conclusion

This prospective multicenter trial showed the beneficial effect of the SGLT2 inhibitor dapagliflozin on LV diastolic functional parameters for T2DM patients with stable HF. Our findings may thus offer a new insight into the management of T2DM patients complicated by HF.

## Abbreviations

A: late diastolic atrial wave velocity
BNP: brain natriuretic peptide
T2DM: type 2 diabetes mellitus
E: early diastolic trans-mitral flow wave velocity
e′: spectral pulsed-wave Doppler-derived early diastolic velocity from the septal mitral annulus
HF: heart failure
HFmrEF: heart failure with mid-range ejection fraction
HFpEF: heart failure with preserved ejection fraction
HFrEF: heart failure with reduced ejection fraction
LAVI: left atrial volume index
LV: left ventricular
LVEF: left ventricular ejection fraction
LVMI: left ventricular mass index
SGLT2: sodium glucose cotransporter type 2
